# Efficient confidence limits for adaptive one-arm two-stage clinical trials with binary endpoints

**DOI:** 10.1186/s12874-017-0297-5

**Published:** 2017-02-06

**Authors:** Guogen Shan, Hua Zhang, Tao Jiang

**Affiliations:** 1 Epidemiology and Biostatistics Program, Department of Environmental and Occupational Health, School of Community Health Sciences, University of Nevada Las Vegas, Las Vegas, 89154 NV USA; 2 School of Computer and Information Engineering, Zhejiang Gongshang University, Hangzhou, 310018 Zhejiang China; 3 Department of Statistics, Zhejiang Gongshang University, Hangzhou, 310018 Zhejiang China

**Keywords:** Adaptive design, Clopper-Pearson approach, Exact one-sided interval, Response rate, Two-stage design

## Abstract

**Background:**

Recently, several adaptive one-arm two-stage designs have been developed by fully using the information from previous stages to reduce the expected sample size in clinical trials with binary endpoints as primary outcome. It is important to compute exact confidence limits for these studies.

**Methods:**

In this article, we propose three new one-sided limits by ordering the sample space based on *p*-value, average response rate at each stage, and asymptotic lower limit, as compared to another three existing sample size ordering approaches based on average response rate. Among the three proposed approaches, the one based on the average response rate at each stage is not exact, and the remaining two approaches are exact with the coverage probability guaranteed.

**Results:**

We compare these exact intervals by using the two commonly used criteria: simple average length and expected length. The existing three approaches based on average response rate have similar performance, and they have shorter expected lengths than the two proposed exact approaches although the gain is small, while this trend is reversed under the simple average criterion.

**Conclusions:**

We would recommend the two exact proposed approaches based on *p*-value and asymptotic lower limit under the simple average length criterion, and the approach based on average response rate under the expected length criterion.

## Background

To assess the activity of a new treatment in a cancer clinical trial, Simon’s two-stage designs [1] are traditionally used among the multi-stage designs. Simon’s two-stage designs can be improved by allowing the second stage sample size to depend on the number of responses observed from the first stage, which is an adaptive two-stage design [2–6]. Recently, Shan et al. [6] developed an adaptive two-stage design that meets the non-increasing relationship between the second stage sample size and the number of responses from the first stage. This is an adaptive one-arm two-stage design that allows the second stage sample size to change with the number of responses observed from the first stage, and the second stage sample size is a non-increasing function of the first stage responses. This sample size monotonic constraint is considered as an intuitive property in adaptive two-stage designs: fewer participants are needed in the second stage when more responses are observed from the previous stage. It should be noted that these adaptive designs [6] often allow early stopping in the first stage due to futility or efficacy, while Simon’s design only allows stopping for futility in the first stage. In these studies, the hypothesis for testing the activity of the new treatment is often one-sided.

Upon completion of an adaptive clinical trial, it is important to provide statistical inference based on the number of responses and the number of participants in each stage. Recently, Zhao et al. [7] proposed a likelihood based approach to construct confidence interval for a study that is designed by Simon’s two-stage method but with unplanned second stage sample size [8, 9]. The likelihood approach was shown to be associated with good performance with regard to coverage probability and coverage bias, but this interval is asymptotic. To guarantee the coverage probability, Shan [10] proposed several new exact confidence intervals based on exact binomial distribution calculation. These intervals are developed for traditional Simon’s two-stage designs whose second stage sample size is considered as fixed regardless the number of responses observed from the first stage as long as it is over the threshold to move to the second stage.

In this article, we consider statistical inference for adaptive two-stage designs whose second stage sample sizes depend on the first stage responses. Adaptive designs are generally flexible and effective as compared to the traditional Simon’s design, however they are often computationally due to many design parameters in the design. With the new adaptive designs being proposed, it is important to develop statistical inference for these designs. To preserve the nominal coverage probability, we propose developing exact one-sided confidence intervals for the response rate in an adaptive two-stage design setting. Confidence intervals are computed by using exact binomial distributions instead of asymptotic distributions. The hypothesis for these trials is often one-sided and the null hypothesis is rejected when a high response rate is observed. For this reason, we focus our approach on exact one-sided lower intervals. The interval is computed by using the approach developed by Clopper and Pearson [11], who proposed the commonly used exact one-sided intervals for a binomial proportion. This approach has to be used in the conjunction with a method to order the sample space.

Multiple approaches have been proposed to order the sample space for multi-stage designs [9, 12–18]. Four orderings were discussed by Jennison and Turnbull [15] after a group sequential design: stage-wise ordering, maximum likelihood estimate (MLE) ordering, likelihood ratio (LR) ordering, and score test (Score) ordering. Lower and upper confidence limits are used in the first ordering. When the outcome is binary, the MLE ordering is equivalent to the ordering by average response rate, which is the number of responses divided by the total sample size in the study. The last two orderings depend on average response rate and the number of sample size from that stage.

In addition to the three aforementioned sample space orderings for a study with binary endpoints, we propose three new methods to order the sample space. The first method is the one based on the response rates from the first and second stages. We consider this is an intuitive method for ordering the sample space based on the information from both stages. However, we find that not all sample points can be ordered by using this method. This leads to the situation in which the nominal coverage probability is not guaranteed. Although the lower limits from the first method are not exact, they can be used as a measurement to order the sample space again. In the second method, each sample point has a unique order number based on the asymptotic lower limit from the first method. The third method uses the *p*-value of each sample point to create a new ordering of the sample space. We find that the ordering based on the p-value has a very interesting relationship with that from the first method.

In “Methods” section, we first introduce the basic settings for adaptive two-stage designs, then propose three new methods to order the sample space. When a study is stopped in the first stage due to either futility or efficacy, their ordering positions are the same in each method. For this reason, we focus on the ordering for sample points when a trial goes to the second stage. We then investigate the coverage probability for each interval. In “Results” section, we compare the performance of the two proposed exact approaches and the three existing approaches with regards to simple average length (AL) and expected length (EL). These approaches are compared by using the completed sample space. In addition to that comparison, we also introduce a new subsample space including the sample points whose second stage response rate is within the confidence interval of the first stage response rate. In other words, if a study’s first stage response rate is very different from its second stage response rate, the study population could be changed (e.g., disease status, gender ratio), and other approaches should be explored for such cases. For this reason, we also utilize this new sample space in the performance comparison among the exact intervals. Finally, we conclude our research with a discussion in “Discussion” section.

## Methods

To test the activity of a treatment in a two-stage design compared to the historical response rate *π*_0_, the hypotheses are often presented as

$$H_{0}: \pi\leq \pi_{0},$$

against the alternative

$$H_{a}: \pi\geq \pi_{1}, $$

where *π*_1_ is the estimated response rate of the new treatment. The null hypothesis is rejected for a high response rate.

Simon’s two-stage design is traditionally used in clinical trials to assess the activity of a new cancer treatment. In Simon’s design, the second stage sample size *n*_2_(*X*_1_) is a constant when a trial goes to the second stage, where *X*_1_ is the number of responses from the first stage. From an adaptive perspective, a clinical trial could be much more flexible and effective when the second stage sample size is allowed to change based on information gathered so far. It is reasonable to assume that *n*_2_(*X*_1_) has a non-increasing relationship with the number of responses observed from the first stage: $n_{2}(X_{1})\geq n_{2}(X_{1}^{'})$ when $X_{1}<X_{1}^{'}$. Shan et al. [6] developed an adaptive optimal two-stage design with the non-increasing sample size relationship respected. This design is referred to be as the AdaptiveS design. The design is given as

$$(n_{1},n_{2}(X_{1}),r(X_{1})), $$

where the number of possible responses out of *n*_1_ participants in the first stage is *X*_1_=0,1,2,…,*n*_1_, and *n*_2_(*X*_1_) and *r*(*X*_1_) are the second stage sample size and the critical value for the study given *X*_1_ responses from the first stage, respectively. In this article, a study can be stopped in the first stage due to futility when *X*_1_≤*r*_1_(*f*) or efficacy when *X*_1_≥*r*_1_(*e*). When the number of responses from the first stage is between *r*_1_(*f*) and *r*_1_(*e*), *r*_1_(*f*)<*X*_1_<*r*_1_(*e*), the trial proceeds to the second stage with an additional *n*_2_(*X*_1_) participants and the final decision is made by comparing the total number of responses (*X*_1_+*X*_2_) and *r*(*X*_1_), where *X*_2_ is the number of responses out of *n*_2_(*X*_1_) participants. The new treatment is considered effective enough to proceed to the next phase when *X*_1_+*X*_2_≥*r*(*X*_1_). Otherwise, the new treatment is not promising for further investigation.

Upon completion of an adaptive clinical trial, confidence interval for the response rate should be computed and reported. The hypothesis for testing the activity of the new treatment is often one-sided, and the confidence interval and the hypothesis testing should be consistent with each other. For this reason, we focus our interest on one-sided lower intervals as the null hypothesis is rejected when a high response rate is observed. When the significance level is *α*, a 1−*α* one-sided interval, (*L*,1], should be computed for statistical inference, where *L* is the 1−*α* lower limit.

The method by Clopper and Pearson [11] (CP) was used to construct exact one-sided intervals for a binomial proportion. It is exact because the coverage probability is guaranteed to be at least 1−*α* and the coverage probability is calculated by using the binomial probabilities, not asymptotic distributions. We extend this approach for the response rate in adaptive two-stage design settings. This approach has to be used with a method to order the sample space, which is often referred to as stochastic ordering. The complete sample space can be divided into three complementary sub-spaces,

$$\Omega=\{G_{1},G_{2},G_{3}\}, $$

where *G*_1_={*X*_1_:0,1,2,…,*r*_1_(*f*)}, *G*_3_={*X*_1_:*r*_1_(*e*),*r*_1_(*e*)+1,…,*n*_1_}, and *G*_2_={(*X*_1_,*X*_2_):*r*_1_(*f*)<*X*_1_<*r*_1_(*e*),*X*_2_≤*n*_2_(*X*_1_)}. Sets *G*_1_ and *G*_3_ contain the sample points where a trial is stopped in the first stage due to futility and efficacy, respectively. Set *G*_2_ represents the sample points that a trial goes to the second stage. It should be noted that set *G*_3_ could be empty in some cases when the optimal adaptive two-stage stops in the first stage only due to futility, which often occurs in cases with a large *π*_0_ and a large difference between *π*_0_ and *π*_1_ as seen in Shan et al. [6]. The lower limits for sample points in set *G*_1_ are the smallest, followed by sample points in set *G*_2_ and set *G*_3_. Within sets *G*_1_ and *G*_3_, the lower limits for the sample points are ordered by the number of their responses, and they are the same as the CP lower limits for a binomial proportion. For sample points in set *G*_2_, the second stage sample size changes as the number of responses from the first stage. For this reason, the second stage sample size should be considered in the sample ordering. We propose ordering the sample points in set *G*_2_ by response rates (RR) in the first stage and the two stages combined,

$$\begin{aligned} L\left(\frac{X_{1}}{n_{1}},\frac{X_{1}+X_{2}}{n_{1}+n_{2}(X_{1})}\right)\leq L\left(\frac{X_{1}^{'}}{n_{1}},\frac{X_{1}^{'}+X_{2}^{'}}{n_{1}+n_{2}\left(X_{1}^{'}\right)}\right)\\ \text{if} \ \ \frac{X_{1}}{n_{1}} \leq \frac{X_{1}^{'}}{n_{1}}\ \ \text{and}\ \ \frac{X_{1}+X_{2}}{n_{1}+n_{2}(X_{1})} \leq \frac{X_{1}^{'}+X_{2}^{'}}{n_{1}+n_{2}\left(X_{1}^{'}\right)}. \end{aligned} $$

This approach is referred to as the RR approach. This ordering is motivated by the *p*-value calculation for a two-stage study. The rejection region includes the extreme outcomes whose first stage and second stage responses are at least as large as the observed data [8–10, 15].

Another stochastic ordering is based on the *p*-value of each sample point. Similar to the RR approach, the *p*-value for sample points in set *G*_1_ is the largest, followed by sample points in set *G*_2_ and *G*_3_. A sample point with a large *p*-value indicates week evidence against the null hypothesis. In other words, it should have a large lower limit. For a sample point (*X*_1_,*X*_2_) in set *G*_2_, its associated *p*-value is calculated as

$${} P(X_{1},X_{2})\,=\,\sum_{(X_{1}^{'},X_{2}^{'})\in \Theta(X_{1},X_{2})}\!b\!\left(\!X_{1}^{'},n_{1},\pi_{0}\!\right)\!b\left(\!X_{2}^{'},n_{2}\left(\!X_{1}^{'}\right),\pi_{0}\!\right), $$

where *b*(…) is the probability density function of a binomial distribution, and *Θ*(*X*_1_,*X*_2_) is the tail area

$${} \begin{aligned} \Theta(X_{1},X_{2})&=\left\{G_{3}\ \text{and}\ \left(X_{1}^{'},X_{2}^{'}\right): \frac{X_{1}}{n_{1}} \leq \frac{X_{1}^{'}}{n_{1}}, \frac{X_{1}+X_{2}}{n_{1}+n_{2}(X_{1})}\right.\\ &\left.\quad\leq \frac{X_{1}^{'}+X_{2}^{'}}{n_{1}+n_{2}\left(X_{1}^{'}\right)} \right\}. \end{aligned} $$

The response rates are used to define the tail area in the *p*-value calculation. Since the *p*-value is used to order the sample space in this approach, we name this approach as the PV approach. Although the *p*-value calculation may not guarantee the type I error rate, it is still a valid measurement to order the sample space. In this approach, we sort the sample points by the *p*-value from smallest to largest. In the PV approach, every sample point has its order number based on its *p*-value, from 1 to the size of the sample space. In the RR approach, two sample points from set *G*_2_ can be ordered only if one sample point belongs to another’s tail area.

Once a stochastic ordering of the sample space is defined, we use the CP method to compute the exact one-sided lower limit as the collection of *π*

1 $$ \left\{\pi:P\big(\Omega_{\varphi}(X_{1},X_{2})|\pi\big)>\alpha\right\},   $$

where *φ* is an approach used to order the sample space (e.g., PV, RR), $\Omega _{PV}(X_{1},X_{2})=\left \{\left (X_{1}^{'},X_{2}^{'}\right):P\right.\left.\left (X_{1}^{'},X_{2}^{'}\right)\right.\left.\leq P(X_{1},X_{2}){\vphantom {(X_{1}^{'},X_{2}^{'}):P}}\right \}$, and $\Omega _{RR}(X_{1},X_{2})=\left \{\left (X_{1}^{'},X_{2}^{'}\right): \left (X_{1}^{'},X_{2}^{'}\right)\right.\left.\in \Theta (X_{1},X_{2}){\vphantom {\left (X_{1}^{'},X_{2}^{'}\right): \left (X_{1}^{'},X_{2}^{'}\right)}}\right \}$. Since the null hypothesis is rejected for a large response rate, we focus on the one-sided lower limit. The proposed approach to compute exact one-sided lower limits can be readily applied to calculate exact upper limits.

### **Theorem 1**

For any given response rate *π*, *P*(*Ω*_*PV*_(*X*_1_,*X*_2_)|*π*)≥*P*(*Ω*_*RR*_(*X*_1_,*X*_2_)|*π*) is always true.

### *Proof*

For sample points from set *G*_1_ and set *G*_3_, it is easy to show that *P*(*Ω*_*PV*_(*X*_1_,*X*_2_)|*π*) is always equal to *P*(*Ω*_*RR*_(*X*_1_,*X*_2_)|*π*). For a given sample point $\left (X_{1}^{'},X_{2}^{'}\right)\in \Omega _{RR}(X_{1},X_{2})$ where sample points (*X*_1_,*X*_2_) and $\left (X_{1}^{'},X_{2}^{'}\right)$ are from set *G*_2_, the relationship between their tail areas is

$$\Theta(X_{1},X_{2}) \supseteq \Theta\left(X_{1}^{'},X_{2}^{'}\right). $$

Thus, the *p*-value of (*X*_1_,*X*_2_) is not less than that of $\left (X_{1}^{'},X_{2}^{'}\right)$: $P(X_{1},X_{2})\geq P\left (X_{1}^{'},X_{2}^{'}\right)$. It follows that the sample point $\left (X_{1}^{'},X_{2}^{'}\right)$ belongs to *Ω*_*PV*_(*X*_1_,*X*_2_). Therefore, *P*(*Ω*_*PV*_(*X*_1_,*X*_2_)|*π*) is always greater than or equal to *P*(*Ω*_*RR*_(*X*_1_,*X*_2_)|*π*) □

From Theorem 1 and the construction of the exact one-sided interval in Eq. (1), we see that the exact one-sided lower limit based on the RR approach is always greater than or equal to that based on the PV approach.

Coverage probability is defined as

2 $${} P\Big(\pi \in (L(X_{1},X_{2}),1]\Big)=P\Big((X_{1},X_{2}):L(X_{1},X_{2})<\pi|\pi\Big).   $$

**Fig. 1:**
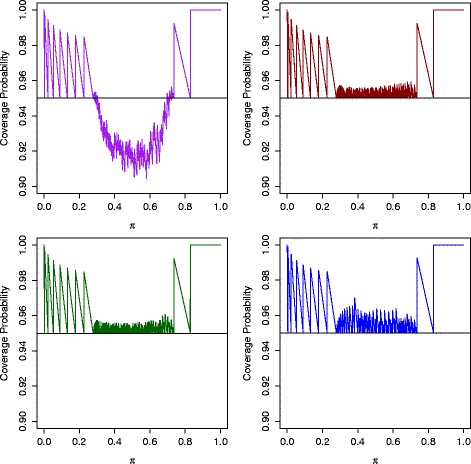
Coverage probability for 95% one-sided lower intervals of the four approaches for the AdaptiveS two-stage design with design parameters (*π*_0_,*π*_1_,*α*,*β*)=(20*%*,40*%*,0.05,0.2). RR approach: *top left*; PV approach: *top right*; RR-A approach: *bottom left*; RR-B approach: *bottom right*

A confidence interval is called exact if *P*(*π*∈(*L*(*X*_1_,*X*_2_),1])≥1−*α* is satisfied for any *π*∈ [ 0,1]. We present the coverage probability plots for the adaptive design with design parameters (*π*_0_,*π*_1_,*α*,*β*)=(20*%*,40*%*,0.05,0.2) [6] in Fig. 1. It can be seen that the PV approach is exact with the coverage probabilities being at least 95%. However, the RR approach is not exact, as the coverage probability could be as low as 90.5% at the nomial level of 95% in this configuration. One reason for the non-exactness of the RR approach is that the sample points can not be completely ordered. To overcome this issuee, we use the non-exact lower limits from the RR approach to order the sample space again. A new stochastic ordering is created by using the calculated limits. This new ordering can be viewed as a two-step ordering because the ordering is generated after the non-exact limit calculation. This approach is referred to as the RR-A approach.

The following three existing approaches to order the sample space have been discussed in the literature [15]. Sample space can be ordered by the average response rate from the study,

$$\frac{X_{1}+X_{2}}{n_{1}+n_{2}(X_{1})}. $$

This approach is referred to as the RR-B approach. It should be noted that this sample size ordering is equivalent to the ordering by using the MLE in a one-sample problem [15]. Another existing sample size ordering is based on the LR method, which is given as

$$\frac{X_{1}+X_{2}}{n_{1}+n_{2}(X_{1})} \sqrt{n_{2}(X_{1})}, $$

named as the RR-LR approach. Similar to the RR-LR approach, Rosner and Tsiatis [14] discussed another ordering based on the score test:

$$\frac{X_{1}+X_{2}}{n_{1}+n_{2}(X_{1})} {n_{2}(X_{1})}. $$

This approach is referred to be as the RR-Score approach. In general, we would expect a significant number of ties when only the response rate is used to order the sample space in traditional two-stage designs. However, the number of ties could be reduced in adaptive design settings as the second stage sample size *n*_2_(*X*_1_) is a non-increasing function of *X*_1_, not a constant.

## Results

**Fig. 2:**
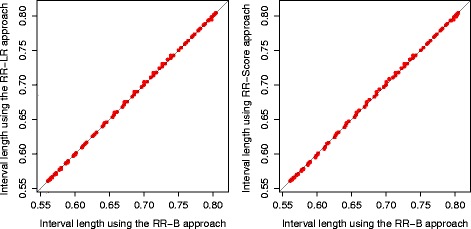
Confidence interval length comparison for each sample point from the adaptive two-stage design with design parameters (*π*_0_,*π*_1_,*α*,*β*)=(30*%*,50*%*,0.05,0.1) when the AdaptiveS design is used. The RR-B approach, the RR-LR approach, and the RR-Score approach are compared

We first compare the performance of the existing three approaches (RR-B, RR-LR, RR-Score) for sample size orderings. The sample space is first ordered by these approaches, then the CP method is used to obtain exact one-sided limits. Figure 2 shows the interval length comparison among the three approach for the adaptive two-stage design with design parameters (*π*_0_,*π*_1_,*α*,*β*)=(30*%*,50*%*,0.05,0.1), where the interval length is defined as [1−*L*(*X*_1_,*X*_2_)] for each sample point (*X*_1_,*X*_2_) from the sample space. The overall average is almost identical for these three approaches. It can be seen from the figure that their interval lengths are very close to each other. Given the simplicity of the RR-B approach, we would recommend that to be used in practice as compared to the RR-LR approach and the RR-Score approach.

We present the coverage probability for the adaptive design with design parameters (*π*_0_,*π*_1_,*α*,*β*)=(20*%*,40*%*, 0.05, 0.2) in Fig. 1. It can be seen that the RR-A approach, the RR-B approach, and the PV approach guarantee the coverage probability while the RR approach does not. For this reason, the RR approach is not going to be included in the following performance comparision. We have illustrated that these three approaches are exact with coverage probability guaranteed. Two criteria, simple average length and expected length, are used to compare the performance of these exact limits. As aforementioned, set *G*_1_ and set *G*_3_ represent a study being stopped in the first stage due to futility or efficacy, respectively. Their lower limits are the same for the three approaches, and actually, they are the exact intervals based on the CP method for a binomial proportion. For this reason, we exclude these sample points in the performance comparison.

The sample space for set *G*_2_ is given as *G*_2_={(*X*_1_,*X*_2_):*r*_1_(*f*)<*X*_1_<*r*_1_(*e*),0≤*X*_2_≤*n*_2_(*X*_1_)}, and the size of this set is

$$M=\sum_{X_{1}=r_{1}(f)+1}^{r_{1}(e)-1}\left(n_{2}(X_{1})+1\right). $$

The simple average length is defined as

$$AL=\frac{\sum\limits_{(X_{1},X_{2})\in G_{2}}\Big[1-L(X_{1},X_{2})\Big]}{M}. $$

**Fig. 3:**
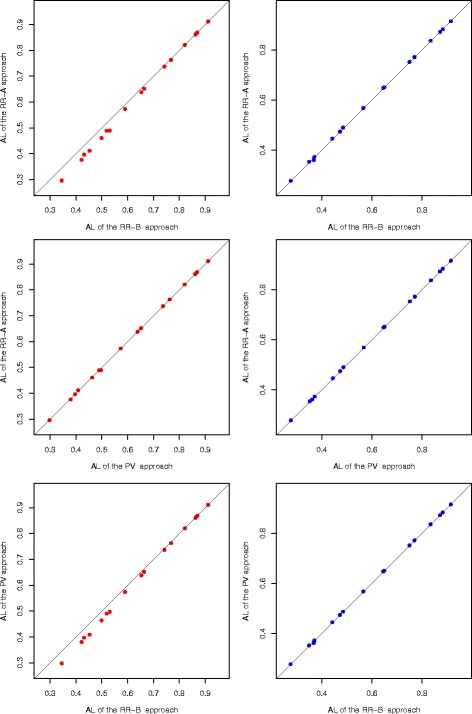
Average length comparison among the three exact approaches (the RR-A approach, the RR-B approach, and the PV approach), for the 16 different configurations with *π*_1_=*π*_0_+20*%* when the AdaptiveS design is used. The sample space *G*_2_ (*left side*) and the subsample space *G*_2_(*C* *I*) (*right side*) are used in computing the 95% one-sided exact lower limits. An approach with a lower average length is preferable

We use 16 different adaptive optimal two-stage designs with *π*_0_ from 5 to 70%, *π*_1_=*π*_0_+20*%*, 80% or 90% power, and *α*=0.05 from Shan et al. [6], in the perfomrance comparison among the three approaches. For each adaptive two-stage design, we first calcualte the exact one-sided lower limits for each sample point in the sample space *G*_2_, then the AL value is computed. The AL comparison among the three approaches for the 16 configurations is presented on the left side of Fig. 3. An approach with a shorter average length is preferable. Thus, the approach on the y-axis performs better than the approach on the x-axis when a point is below the diagnoal line. Each point in the plot represents the AL values for an adaptive design. It can be seen that the RR-A approach and the PV approach often have shorter average lengths than the RR-B approach, and the RR-A approach and the PV approach are generally comparable. We also investigate another 16 configurations with *π*_1_=*π*_0_+15*%* as in Shan et al. [6], and we observe similar conclusions. We compute the overall average of AL values for these 32 configurations. The RR-A approach has the shortest overall average length (0.6057), followed by the PV approach (0.6069), and the RR-B approach (0.6273).

It should be noted that some sample points in set *G*_2_ may not be practically possible, for example, a sample point with a relatively very low or high estimated second stage response rate as compared to the first stage response rate. From a practical perspective, it would be reasonable to expect a study with similar response rates from each stage. For this reason, we create a new subset of *G*_2_ to compare the perform again, and the new subset includes the sample points whose second stage response rates are within the 95% confidence interval of their first stage response rates. The confidence interval of the first stage response rate is computed by the function *exactci* from R package *PropCIs* [19]. The CP method is used in the function *exactci* to obtain exact two-sided intervals for a binomial proportion. The new subset of *G*_2_ is defined as *G*_2_(*C* *I*)

$$\begin{aligned} G_{2}(CI)&=\left\{(X_{1},X_{2}): r_{1}(f)<X_{1}<r_{1}(e), 0\leq X_{2}\leq n_{2}\right.\\ &\qquad\left.(X_{1}), X_{2}\in CI(X_{1},n_{1},95\%)\right\}, \end{aligned} $$

where *C* *I*(*X*_1_,*n*_1_,95*%*) is the 95% exact two-sided interval for the response rate *X*_1_/*n*_1_. The simple average length for sample points in set *G*_2_(*C* *I*) is computed as

$$AL=\frac{\sum\limits_{(X_{1},X_{2})\in G_{2}(CI)}\Big[1-L(X_{1},X_{2})\Big]}{M(CI)}, $$

where *M*(*C* *I*) is the size of sample space *G*_2_(*C* *I*). The three approaches are also compared for the 16 adaptive designs when the sample space is *G*_2_(*C* *I*), in Fig. 3. It should be noted that *G*_2_(*C* *I*) is the same for the three approaches as it only depends on the first stage response rate, not on the approach. After removing the non-practical sample points, the three approaches have very close average lengths. By using the total of 32 configurations as aforementioned, the overall average length when using the subsample space *G*_2_(*C* *I*) is 0.6018, 0.6028, and 0.6038 for the RR-A approach, the PV approach, and the RR-B approach, respectively. It suggests that the RR-A approach has the best performance when *G*_2_(*C* *I*) is considered. We include the sample points whose second stage response rate are within the 95% confidence interval of their first stage response rates. If the current considered confidence level of 95% is decreased to 80% or increased to 97.5%, we observe similar results. When it is increased to 99% with more sample points in the sample space, the results are similar to these observed from the case when *G*_2_ is the sample space. When the confidence level is increased to 100%, the sample space *G*_2_(*C* *I*) is the same as *G*_2_.

**Fig. 4:**
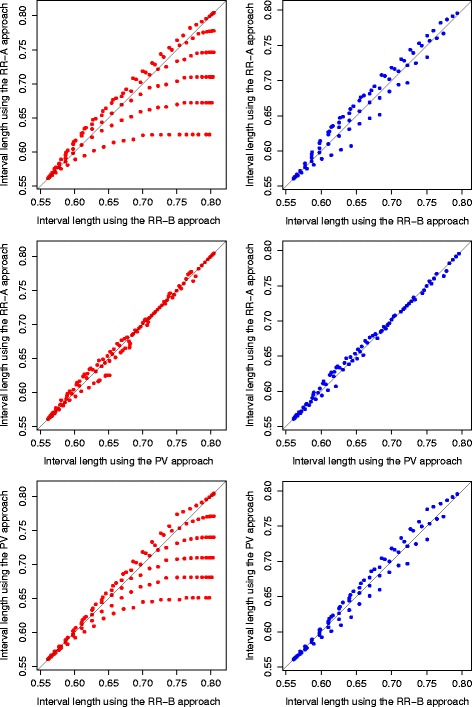
Confidence interval length comparison for each sample point from the adaptive two-stage design with design parameters (*π*_0_,*π*_1_,*α*,*β*)=(30*%*,50*%*,0.05,0.1) when the AdaptiveS design is used. The RR-A approach, the RR-B approach, and the PV approach are compared on the left side when the sample space *G*_2_ (*left side*) is used, and on the right side when the subsample space *G*_2_(*C* *I*) (*right side*) is used

In addition to the average length comparison among the three approaches, we also present the interval length comparison between all the sample points, [1−*L*(*X*_1_,*X*_2_)], in Fig. 4 for the adaptive two-stage design with design parameters (*π*_0_,*π*_1_,*α*,*β*)=(30*%*,50*%*,0.05,0.1). When all sample points from *G*_2_ are considered, the RR-B approach has longer lengths than the other two approaches in general. However, the three approaches are similar to each other for sample points from the subset space *G*_2_(*C* *I*). The advantage of the RR-A approach and the PV approach over the RR-B approach is mostly due to the sample points that are not practically possible. The RR-A approach and the PV approach are comparable under either sample space. Similar results are observed from other configurations, and other existing adaptive two-stage designs that do not meet the monotonic sample size property [20].

**Fig. 5:**
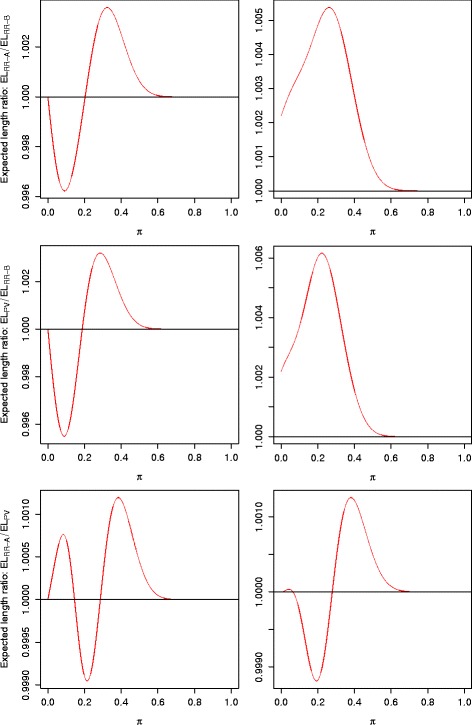
Expected length comparison among the RR-A approach, the RR-B approach, and the PV approach, for the adaptive two-stage design with design parameters (*π*_0_,*π*_1_,*α*,*β*)=(20*%*,40*%*,0.05,0.1) when the AdaptiveS design is used. The sample space *G*_2_ (*left side*) and the subsample space *G*_2_(*C* *I*) (*right side*) are used to compute the expected lengths

Under the simple average length criterion, each sample point’s interval length in the sample space is weighted equally. It is also important to compare the performance of exact limits by using the expected length criterion. Under this criterion, each sample point’s interval length is weighted by its associated probability at the response rate *π*. Thus, the EL is a function of *π*. Specifically, the EL is defined as

$${} EL(\pi\!)\,=\,\sum_{(X_{1},X_{2})\in G_{2}}\!\![\!1-L(X_{1},X_{2})]b(X_{1},n_{1},\pi\!)b(X_{2},n_{2}(X_{1}\!),\pi\!), $$

where *b*(*X*_1_,*n*_1_,*π*)*b*(*X*_2_,*n*_2_(*X*_1_),*π*) is the probability of the observed data (*X*_1_,*X*_2_) with *n*_1_ and *n*_2_(*X*_1_) as the first stage and the second stage sample sizes. For a given *π*, we first compute the *E* *L*_*RR*−*A*_(*π*), *E* *L*_*RR*−*B*_(*π*), and *E* *L*_*PV*_(*π*). Their differences are generally small, especially in cases where *π* is near the boundary. For this reason, we use their ratios to compare them. Figure 5 displays the EL ratio plots for the adaptive two-stage design with design parameters (*π*_0_,*π*_1_,*α*,*β*)=(20*%*,40*%*,0.05,0.1). In general, these three approaches are comparable with regards to the EL criterion, with the EL ratio between 99.5% and 100.6%. When set *G*_2_ is the sample space, the RR-B approach has a shorter expected length than the other two approaches when *π*>20*%*. When the sample space *G*_2_(*C* *I*) is used, the RR-B appraoch generally performs better than the RR-A approach and the PV approach, although the gain is small. The RR-A approach has a shorter expected length than the PV approach when *π* is near *π*_0_=20*%*. Similar results are observed when other adaptive designs are used.

### Example

Since the considered adaptive two-stage designs are relatively new, we do not expect to find a real data set to be used. For this reason, we assume that a study is designed by using the AdaptiveS design with design parameters (*π*_0_,*π*_1_,*α*,*β*)=(30*%*,50*%*,0.05,0.1). In the first stage, *X*_1_=11 responses are observed among the *n*_1_=22 patients. Then, the required number of patients is *n*_2_(*X*_1_)=35 for the second stage. At the end of the study, we assume that *X*_2_=13 responses are observed from the second stage. Thus, the total number of responses is 11+13=24, and the average response rate is estimated as 24/(22+35)=42.1*%*. The 95% lower limit for the response rate is calcualted as 0.320 for the PV approach, 0.325 for the RR-A approach, 0.317 for the RR-B approach, 0.317 for the RR-LR approach, 0.317 for the RR-Score approach, and 0.344 for the RR approach. It can be seen that the RR-B approach, the RR-LR approach, and the RR-Score approach have very similar lower limits, and they are smaller than others. The RR approach has the largest lower limit among these approaches.

We also use this example to explain the reason why the RR approach is not exact. In the RR approach, the rejection region includes the extreme outcomes whose first stage and second stage responses are at least as large as the observed data. If the observed data is *X*_1_=11 and *X*_2_=13, then the sample point (*X*_1_=12,*X*_2_=11) does not belong to the observed data’s rejection region since (12+11)/(22+35)<(11+13)/(22+35). By using the RR approach, the calculated 95% lower limit for (*X*_1_=12,*X*_2_=11) is 0.362, which is larger than that of the observed data’s. It follows that

$${} (X_{1}\!=12, X_{2}=\!11)\!\notin \{(X_{1},X_{2}):L(X_{1},X_{2})<\pi|\pi=0.343\}. $$

Thus, the sample point (*X*_1_=12,*X*_2_=11) does not belong to the observed data’s confidence set, neither. Therefore, the coverage probability at *π*=0.343 could be less than the nominal level. In other words, in a two-stage design, inverting the *p*-value function is exact only when the sample space can be ordered completely by a test statistic or a measurement.

## Discussion

In addition to the proposed confidence interval for adaptive two-stage designs to make statistical inference, unbiased response rate estimate and exact *p*-value calculation are two important future research topics. Due to the multi-stage nature of the design, the naive estimate that divides the total number of responses by the total number of participants, is biased. Jung and Kim [21] proposed an unbiased response rate estimate for traditional non-adaptive two-stage designs where the second stage sample size is a constant for a trial proceeding to the second stage. In adaptive two-stage design settings, the second stage sample size is a non-increasing function of the responses from the first stage. We consider this as future work to further develop an unbiased response estimate and exact *p*-value calculations in adaptive two-stage design settings.

## Conclusions

Multiple adaptive two-stage designs have been proposed for use in practice, but these is limited research on analyzing the data once an adaptive study is finished. This article proposes three approaches to construct one-sided lower limits. Among these three new approaches, two approaches guarantee coverage probability. We compare these two exact intervals with another three existing approaches with regards to the two commonly used criteria: simple average length and expected length. The RR-A approach and the PV approach have similar performance with regards to these two criteria, although the RR-A approach performs slightly better than the PV approach under the AL criterion. The RR-B approach has a slightly longer average length than the other two approaches when all sample points are used in the calculation, and their difference becomes negligible when the subsample space *G*_2_(*C* *I*) is used. In addition, the RR-B approach generally has a shorter expected length than the RR-A approach and the PV approach by using the sample space *G*_2_(*C* *I*) [22–25].
